# Experimental Study on Laser-Induced Damage Performance of CO_2_ Laser-Polished Fused Silica Components

**DOI:** 10.3390/mi16121400

**Published:** 2025-12-12

**Authors:** Ting Tan, Qiao Xu, Shengfei Wang, Jin Zhuo, Feng Geng, Zhichao Liu, Huiliang Jin, Xiangfeng Wang, Hongjun Liu, Qinghua Zhang

**Affiliations:** Research Center of Laser Fusion, China Academy of Engineering Physics, Mianyang 621900, Chinarobertwsf@sina.com (S.W.);

**Keywords:** fused silica, CO_2_ laser polishing, damage performance

## Abstract

Enhancing the laser-induced damage resistance (LIDR) of fused silica components is crucial for improving the overall performance of large-scale laser systems. However, traditional optical manufacturing techniques, based on contact processing principles, inevitably lead to the generation of defects such as scratches and contamination during processing, which severely limit the improvement of component LIDR. To address this issue, this study employs a CO_2_ laser to polish ground fused silica samples. Through experimental analysis of the influence of pre-treatment processes and laser processing parameters on the damage performance of the laser-polished samples, an optimized laser polishing process scheme was obtained. Fused silica samples processed using the optimized laser polishing scheme were compared with those treated by conventional polishing and etching in terms of damage performance. The results indicate that, compared to conventional polished and etched samples, the laser-polished samples exhibited an approximately 20% increase in damage threshold and a 76.4%~90.8% reduction in damage density. The damage performance of the laser-polished samples was significantly superior to that of conventional polished and etched samples. Through experimental analysis, this paper obtained an optimized laser polishing process scheme and confirmed the definitive role of CO_2_ laser polishing in enhancing the LIDR of fused silica samples, providing technical support for the development of next-generation optical manufacturing technologies.

## 1. Introduction

Fused silica material, as a key choice for the high-fluence sections of large-scale laser systems, is widely used in manufacturing optical components such as windows, gratings, and lenses due to its excellent optical properties [1]. Improving the laser-induced damage resistance of fused silica components has become essential for enhancing the load capacity of laser facilities and optimizing the overall performance of large-scale laser systems. However, traditional optical manufacturing processes are contact-based. While achieving surface polishing, these methods inevitably generate defects like contamination, scratches, and cracks onto the component surface [2]. During the operation of large-scale laser systems, these surface defects on fused silica components absorb laser energy, leading to component damage at fluences far below their intrinsic threshold [3,4,5,6], severely affecting component performance and service life. Currently, laser-induced damage initiated at surface defects is a bottleneck limiting the improvement of the load capacity of large-scale laser systems. Enhancing the laser-induced damage resistance of fused silica optical components is a significant research topic for extending optical component lifespan, reducing operational costs of large laser systems, and promoting the further development of laser technology [7].

Due to the strong absorption characteristics of fused silica material at the CO_2_ laser wavelength of 10.6 μm, researchers have proposed using CO_2_ lasers to achieve the fusion of subsurface cracks and smoothing of the surface profile of fused silica components. This technique utilizes CO_2_ laser irradiation to rapidly heat an extremely thin surface layer of the fused silica component to a molten state. Through the combined action of surface tension and the Marangoni effect, micro-flow and molten healing of the surface material are achieved, thereby realizing non-contact processing to fuse subsurface cracks, smooth the surface profile, and reduce surface roughness [8,9].

In recent years, domestic and international scholars have conducted extensive research on CO_2_ laser polishing of fused silica, primarily focusing on the influence of process parameters on polishing effects [10,11,12,13,14,15,16], surface optimization treatment and polishing mechanism analysis [17,18,19,20,21], and local damage repair [22,23,24,25,26]. Numerous experimental results show that through optimization of process parameters, laser polishing can reduce the surface roughness of fused silica components from the sub-micron level to the nanometer level, effectively repair micron-scale scratches, heal subsurface cracks, and obtain smooth component surfaces. However, the current focus remains largely on the improvement of surface morphology and local damage repair of fused silica components, with systematic research on the key performance indicator—laser-induced damage resistance—still being insufficient.

This study analyzed the influence of CO_2_ laser polishing pre-treatment processes and laser processing parameters on the damage performance of laser-polished fused silica samples, obtaining an optimized laser polishing process scheme. A comparative analysis of the damage performance between samples processed with the optimized laser polishing and those treated with conventional polishing and etching was conducted, verifying the superior damage performance of the laser-polished samples and confirming the definitive role of CO_2_ laser polishing in enhancing the laser-induced damage resistance of fused silica samples.

## 2. Materials and Methods

### 2.1. Materials

The fused silica samples used in this experiment were made of JGS1 fused silica glass. The components were ground using W10 loose abrasive grinding with abrasive particle sizes ranging from 5 μm to 10 μm. Unless otherwise specified, the component dimensions were φ50 mm × 5 mm.

### 2.2. Methods

**Figure 1:**
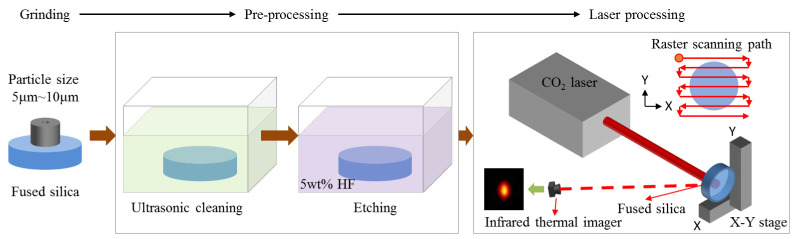
Laser polishing process flowchart.

Laser polishing primarily utilizes the strong absorption characteristic of fused silica material at the CO_2_ laser wavelength, causing the surface layer material to melt and undergo micro-flow through the deposition of laser energy, thereby achieving crack healing, surface smoothing, and damage performance enhancement. The laser polishing process flow is shown in Figure 1, mainly comprising pre-treatment processes and laser processing. Pre-treatment includes ultrasonic cleaning and etching, with the etching method being 5 wt% HF acid etching. Laser processing primarily involves CO_2_ laser scanning processing using a raster scanning pattern. Process temperature monitoring was performed using an infrared thermal imager. Surface roughness measurement of processed components was conducted using an atomic force microscope (AFM). Observation of subsurface crack fusion effect was carried out using a scanning electron microscope (SEM). Damage pit morphology was observed using a confocal microscope. Damage threshold testing was performed using a damage test platform with a wavelength of 355 nm, pulse width of 3 ns, and spot diameter of 1 mm, following the ISO 1-on-1 test method. Damage density testing was conducted using a damage test platform with a wavelength of 351 nm, pulse width of 5 ns, and spot diameter of 8.4 mm.

## 3. Results and Discussion

### 3.1. Influence of Pre-Treatment Processes

Pre-treatment processes mainly include ultrasonic cleaning and HF acid etching. This study first analyzed the effects of cleaning method, cleaning water quality (the test results are shown in Table 1), and pre-etching on the laser-induced damage threshold (LIDT) of laser-polished fused silica samples.

**Figure 2:**
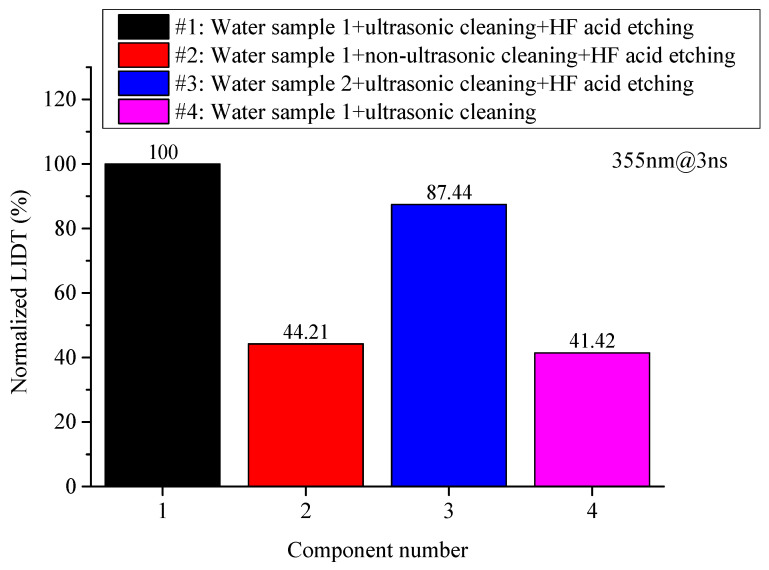
Comparison results of the normalized laser-induced damage thresholds for laser-polished samples under different pre-treatment conditions.

**Figure 3:**
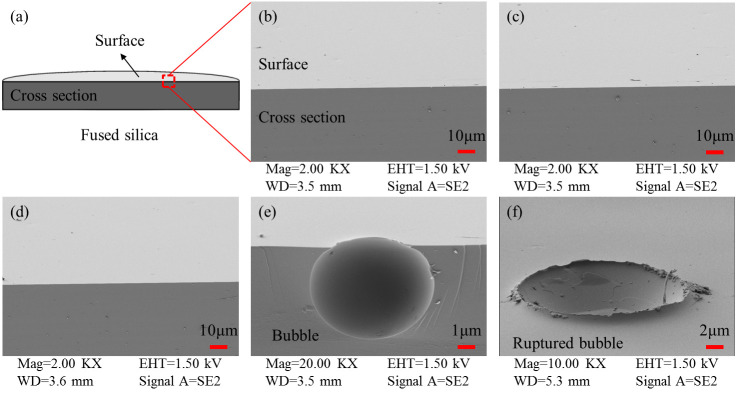
SEM images of subsurface crack fusion effect in laser-polished samples. (**a**) Observation schematic; (**b**,**c**) Sample #1: pre-etched before 2000 °C laser processing; (**d**–**f**) Sample #4: non-etched before 2000 °C laser processing.

Figure 2 compares the normalized laser-induced damage thresholds for laser-polished fused silica samples under different pre-treatment conditions. The results indicate that regarding the cleaning method, the damage threshold of sample #2 subjected to “non-ultrasonic cleaning (water jet rinsing)” decreased by approximately 56% compared to sample #1 subjected to “ultrasonic cleaning,” showing a significant threshold reduction. This demonstrates that rigorous cleaning can remove surface contaminants to the greatest extent, favoring the improvement of damage performance during laser polishing. Therefore, in practical applications, strict ultrasonic cleaning of components must be performed before laser processing to ensure the cleanliness of components entering the process and avoid the impact of incomplete cleaning on the damage performance of processed components. Regarding cleaning water quality, the damage threshold of sample #3 cleaned with “Water Sample 2”, which has higher metal impurity content, decreased by approximately 13% compared to sample #1 cleaned with “Water Sample 1”, which has higher cleanliness. Although the decrease is relatively smaller, the downward trend remains evident. Combined with the influence pattern of the cleaning method on the damage performance of laser-processed samples, it can be seen that surface contamination significantly affects the final damage performance of components. The cleaning process should strive to improve surface cleanliness, including using clean water and rigorous cleaning procedures. Regarding pre-etching, the damage threshold of sample #4 without pre-etching decreased by approximately 59% compared to sample #1 with pre-etching, indicating a substantial threshold reduction. Simultaneously, the subsurface crack fusion effect of samples #1 and #4 were observed using SEM, as shown in Figure 3. From Figure 3, it can be seen that after laser polishing, compared to sample #1, sample #4 without pre-etching treatment tends to have residual bubbles on the surface, with bubble morphologies ranging from complete spheres to semi-ruptured spheres, and bubble diameters reaching the micron scale, failing to achieve complete fusion of subsurface cracks. This indicates that appropriate pre-etching, while removing surface hydrolyzed layer contamination, can effectively expose subsurface cracks in fused silica components, promote the molten healing efficiency, and is beneficial for enhancing the damage performance of CO_2_ laser-polished samples.

**Figure 4:**
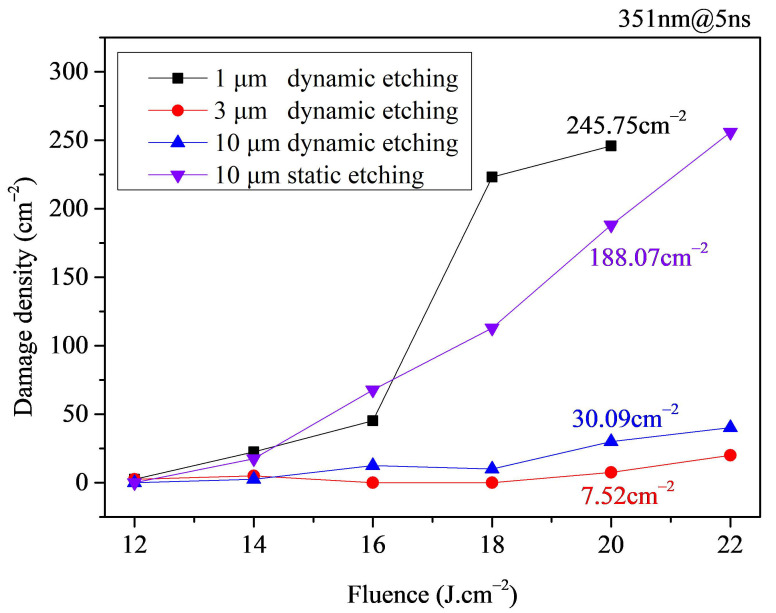
Damage density test results for laser-polished samples under different pre-etching conditions.

Moreover, the effects of etching method and etching depth on the damage density of laser-polished fused silica samples were further analyzed, with results shown in Figure 4. The results indicate that regarding the etching method, the damage density of components subjected to 10 μm static etching can be 5 to 10 times that of components subjected to 10 μm dynamic etching, proving that dynamic etching can minimize the generation of re-deposited material during etching, favoring the reduction in damage density in laser-polished samples. Regarding etching depth, components subjected to 1 μm dynamic etching exhibited the highest damage density, reaching over 10 times that of components subjected to 3 μm and 10 μm dynamic etching at some fluence levels. Meanwhile, the damage density of components subjected to 10 μm dynamic etching was significantly higher than that of components subjected to 3 μm dynamic etching; at some fluence levels, the damage density of the 10 μm dynamically etched components could be over 2 times that of the 3 μm dynamically etched components. This suggests that the etching depth should be appropriate—neither too deep nor too shallow. When the etching depth is too shallow, the surface hydrolyzed layer becomes difficult to remove completely. On the contrary, if the etching depth is too deep, etching deposition on the component surface increases, leading to secondary contamination of the surface.

From the above results, it can be concluded that controlling surface contamination plays a crucial role in enhancing the laser-induced damage resistance of fused silica samples. Furthermore, as laser polishing is a non-contact processing technique, the laser processing itself does not introduce new contamination. Therefore, the key to contamination control for laser-polished samples lies in the pre-treatment process. To remove original contamination while minimizing the generation of new contaminants and obtain laser-polished fused silica samples with optimized damage performance, the pre-treatment process should maintain standard cleaning procedures, appropriate etching, and a clean water environment.

### 3.2. Influence of Laser Processing Parameters

During the laser processing stage, the surface layer material of the fused silica component melts and undergoes micro-flow through the deposition of laser energy, achieving crack healing, surface smoothing, and damage performance enhancement. This process involves many influencing factors, but its essence is the molten healing of the softened layer material in the irradiated area. The molten state is directly related to temperature. Therefore, in the laser processing stage, this study primarily focuses on the experimental analysis of the influence of the core factor—processing temperature—on damage performance.

**Figure 5:**
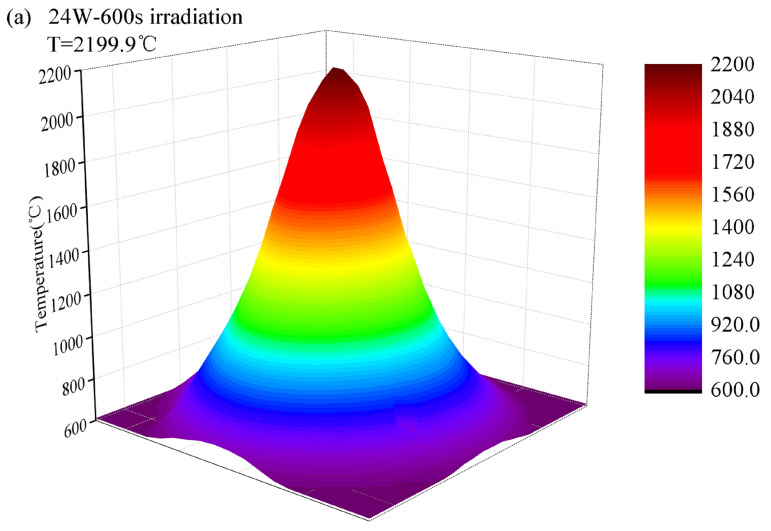

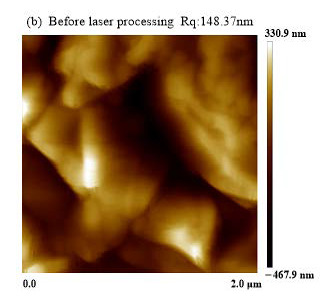

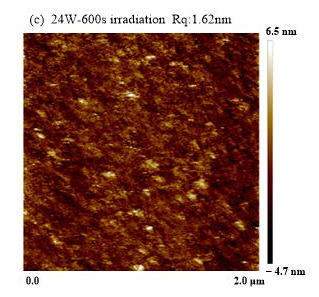
Schematic diagram of thermal imager and AFM measurement results. (**a**) Temperature distribution; (**b**) AFM image before laser processing; (**c**) AFM image after laser processing at 24 W for 600 s.

First, to verify the effect of processing temperature on the crack fusion effectiveness during laser processing, an infrared thermal imager and an atomic force microscope were used to measure the surface temperature and roughness of fused silica components under localized irradiation with different laser powers. Figure 5 shows the surface temperature and roughness measurement results of the irradiated area on a fused silica component under localized irradiation with a 24 W CO_2_ laser for 600 s.

**Figure 6:**
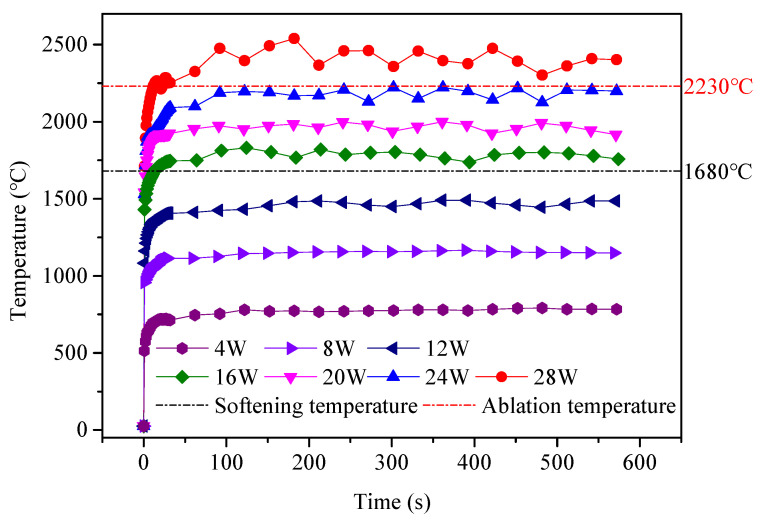
Variation in maximum temperature in irradiated area with irradiation time under different laser power localized irradiation conditions.

**Figure 7:**
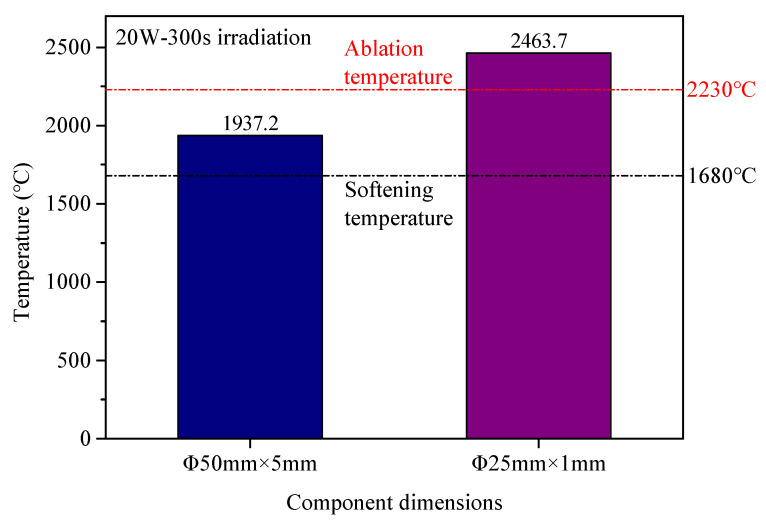
Difference in maximum temperature in irradiated area for components of different sizes.

Figure 6 shows the variation in the maximum surface temperature in the irradiated area of the fused silica component with irradiation time under different laser power localized irradiation conditions. From Figure 6, it can be seen that when the laser power is constant, as the irradiation time increases, the maximum surface temperature in the irradiated area rapidly increases within approximately 1 min, then reaches an equilibrium temperature and remains relatively stable. During laser scanning processing, the scanning speed should be controlled so that the scanning temperature approaches this equilibrium temperature, thereby achieving a relatively stable laser scanning processing effect. When the irradiation time is constant and sufficiently long, the equilibrium surface temperature in the irradiated area increases with increasing laser power. Furthermore, influenced by the stability of the laser output power, as the laser power increases, the stability of the output power deteriorates, leading to increased fluctuation of the equilibrium temperature under corresponding conditions. Figure 7 shows the measurement results of the maximum temperature in the irradiated area on the surfaces of φ50 mm × 5 mm component and φ25 mm × 1 mm component under localized irradiation with a 20 W CO_2_ laser for 300 s. From Figure 7, it can be seen that under the same irradiation conditions, the maximum surface temperature of φ25 mm × 1 mm component is about 500 °C higher than that of φ50 mm × 5 mm component. Different-sized components exhibit significant surface temperature differences under identical irradiation conditions. This indicates that for fused silica samples with identical surface defect states but different component sizes to achieve the same crack healing and damage performance enhancement effect, direct control of the laser fusion temperature should be implemented rather than indirect control of laser irradiation parameters. To ensure the quality of laser polishing, the processing temperature must be precisely maintained between the material’s softening temperature (1680 °C) and ablation temperature (2230 °C) [27]. The results in Figure 6 indicate that for the φ50 mm × 5 mm component, controlling the laser power within the range of 16 W~24 W keeps the temperature within this desirable interval. In contrast, for the φ25 mm × 1 mm component, a laser power of 20 W raises the processing temperature above the material’s ablation temperature. Hence, the laser power must be further reduced to maintain the processing temperature within the corresponding range.

**Figure 8:**
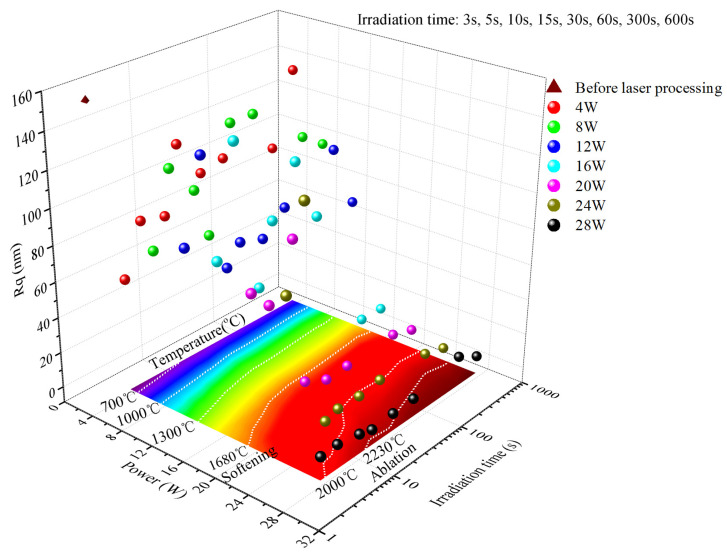
Variation in surface roughness at irradiation center with temperature at irradiation center under different laser power localized irradiation conditions.

Figure 8 shows the variation in surface roughness in the irradiated area with the maximum temperature in that area for ground fused silica surfaces under localized irradiation with different laser powers. Combined with Figure 6 and Figure 8, it can be seen that as the laser power increases, the temperature in the irradiated area gradually increases. If the laser power is too low (<16 W), the equilibrium surface temperature in the irradiated area remains low, preventing material softening and melting; regardless of irradiation duration, the surface roughness cannot be reduced. If the laser power is sufficiently high (>16 W), corresponding to an equilibrium temperature above the softening temperature, a gradual decrease in surface roughness with increasing irradiation time and temperature can be observed. This demonstrates that the direct factor for crack molten healing is the temperature in the irradiated area. When this temperature reaches the softening temperature of fused silica, the surface material begins to melt and undergo micro-flow, leading to the gradual optimization of surface roughness. Furthermore, the higher the temperature in the irradiated area, the faster the roughness optimization. Conversely, if the laser power is too low, and the corresponding equilibrium temperature never reaches the softening temperature, the roughness cannot be optimized regardless of irradiation time. In contrast to previous studies [28,29,30], this work not only confirms the influence of laser power on the surface roughness of laser-polished components, but also further reveals the critical role of temperature parameters. It thereby identifies the processing temperature as the direct controlling factor for optimizing surface roughness in laser polishing.

Combining Figure 6, Figure 7 and Figure 8, it is evident that for laser polishing effectiveness, the laser polishing processing temperature is the direct influencing factor, while laser power, component size, etc., are indirect factors that affect the laser polishing outcome by influencing the irradiated area temperature. Therefore, this study analyzes the influence of processing temperature on the damage performance of laser-polished samples. All samples underwent the pretreatment procedure of Sample #1 (water sample 1 + ultrasonic cleaning + HF acid etching), a protocol identified as optimal in prior analysis (Section 3.1).

**Figure 9:**
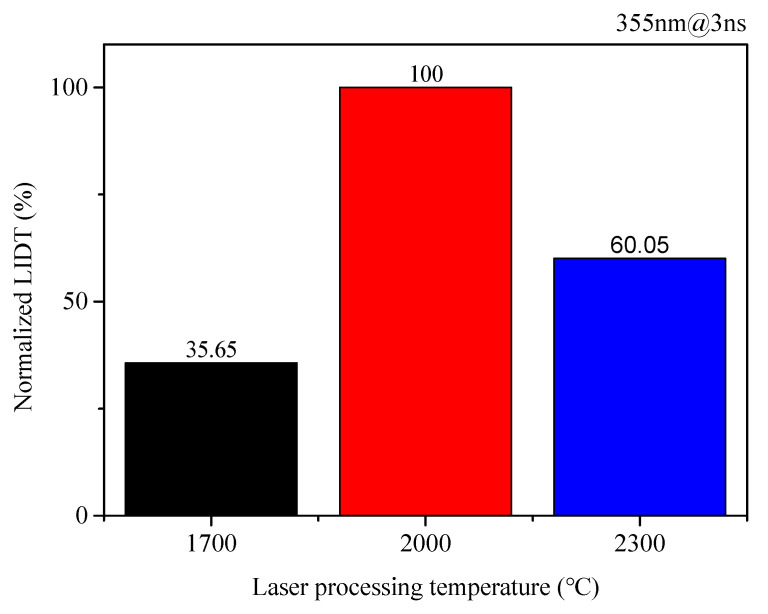
Comparison results of the normalized laser-induced damage thresholds for laser-polished samples under different processing temperature conditions.

**Figure 10:**
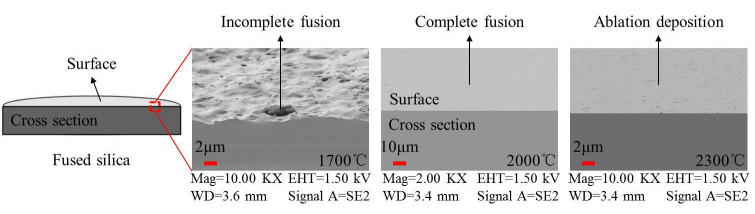
SEM images of subsurface crack fusion effect in laser-polished samples under different processing temperature conditions.

The comparison results of the normalized laser-induced damage thresholds for laser-polished fused silica samples processed at thermal fusion temperatures of 1700 °C, 2000 °C, and 2300 °C are presented in Figure 9, with the corresponding SEM images of subsurface crack fusion effect shown in Figure 10. Combining Figure 9 and Figure 10, it can be seen that at 1700 °C, the processing temperature is close to the softening temperature; the surface profile of the processed sample begins to smooth, but subsurface cracks remain largely unfused. The damage threshold decreases by approximately 65% compared to samples processed at 2000 °C. At 2000 °C, the processing temperature is above the softening temperature but below the ablation threshold; the subsurface cracks in the processed samples are basically fused, and the sample damage threshold is significantly higher than that of samples processed at 1700 °C and 2300 °C. At 2300 °C, the processing temperature is close to the ablation temperature; the processed sample surface shows ablation deposition, with evaporated material adhering to the surface and being difficult to remove completely, adversely affecting the sample’s damage performance. The damage threshold decreases by approximately 40% compared to samples processed at 2000 °C. This demonstrates that the improvement in damage performance of laser-polished samples is directly related to the fusion state of subsurface cracks in the fused silica samples, and the effectiveness of subsurface crack molten healing is directly affected by the processing temperature. Therefore, during laser processing, an appropriate laser processing temperature must be selected to ensure effective fusion of subsurface cracks, thereby achieving the optimal damage performance enhancement.

**Figure 11:**
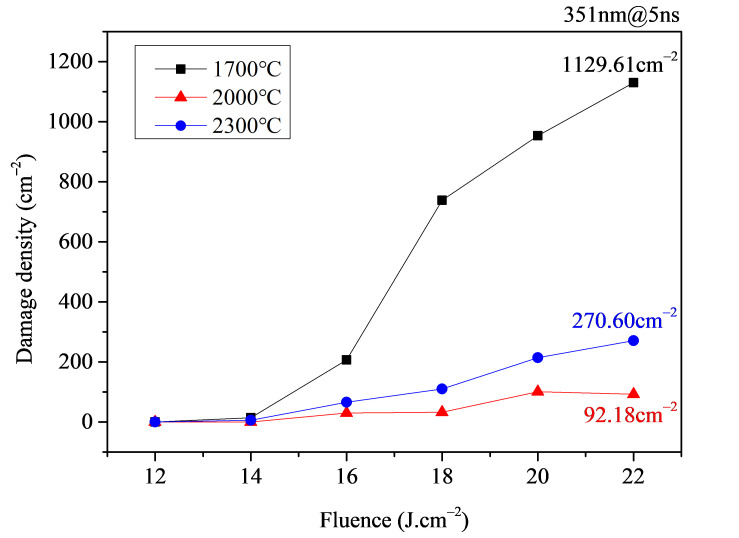
Damage density test results for laser-polished samples under different processing temperature conditions.

Figure 11 compares the test results of damage density under processing temperatures of 1700 °C, 2000 °C, and 2300 °C. From the figure, it can be seen that as the laser processing temperature increases, the trend in damage density change is opposite with that of the damage threshold, generally showing an initial decrease followed by an increase. Specifically, under the 1700 °C processing condition, due to the relatively low processing temperature, subsurface cracks cannot be effectively fused, resulting in the highest damage density. Under the 2000 °C condition, subsurface cracks are completely fused and the surface material does not undergo ablation, resulting in the lowest damage density. The difference between these two can exceed a factor of 10. Under the 2300 °C processing condition, the processing temperature is close to the ablation temperature, and the damage performance is affected by ablation deposition on the component surface, leading to an increase in damage density by approximately 2~3 times compared to samples processed at 2000 °C. This further confirms the critical role of effective subsurface crack fusion in enhancing the damage performance of laser-polished samples.

From the above results, it can be concluded that the key to improving the damage performance of laser-polished samples during laser processing lies in achieving effective fusion of subsurface cracks without inducing ablation. The thermal fusion temperature, as the direct factor affecting crack fusion effectiveness, is a crucial parameter influencing the laser polishing outcome. To achieve the optimal damage performance enhancement, an appropriate thermal fusion temperature should be selected during laser processing, ensuring maximum fusion of subsurface cracks while avoiding ablation.

### 3.3. Comparison with Conventional Polished and Etched Samples

**Figure 12:**
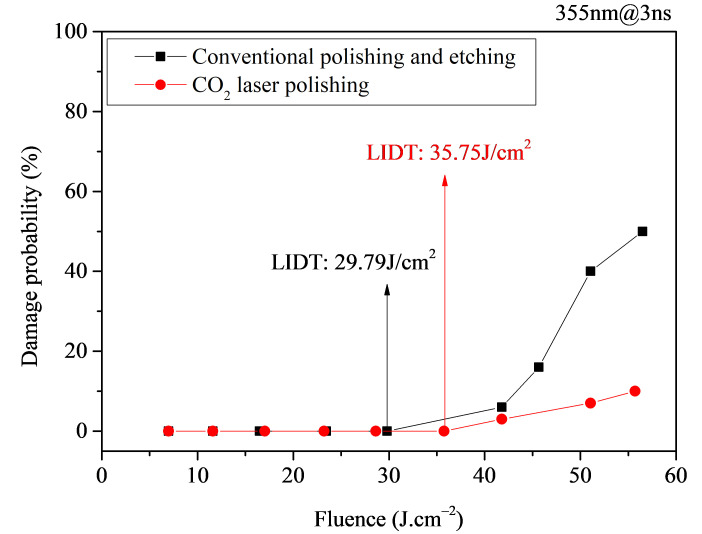
1-on-1 laser damage test results for laser-polished samples and conventional polished and etched samples.

**Figure 13:**
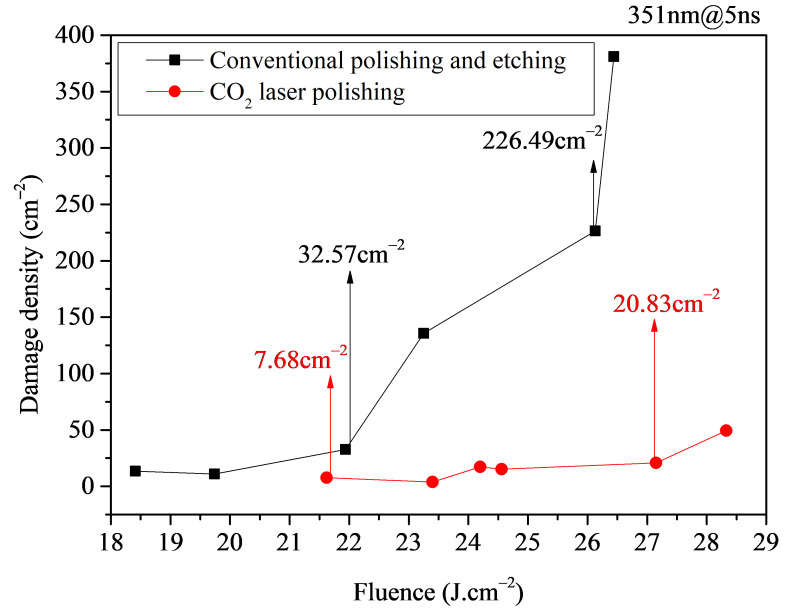
Damage density test results for laser-polished samples and conventional polished and etched samples.

**Figure 14:**
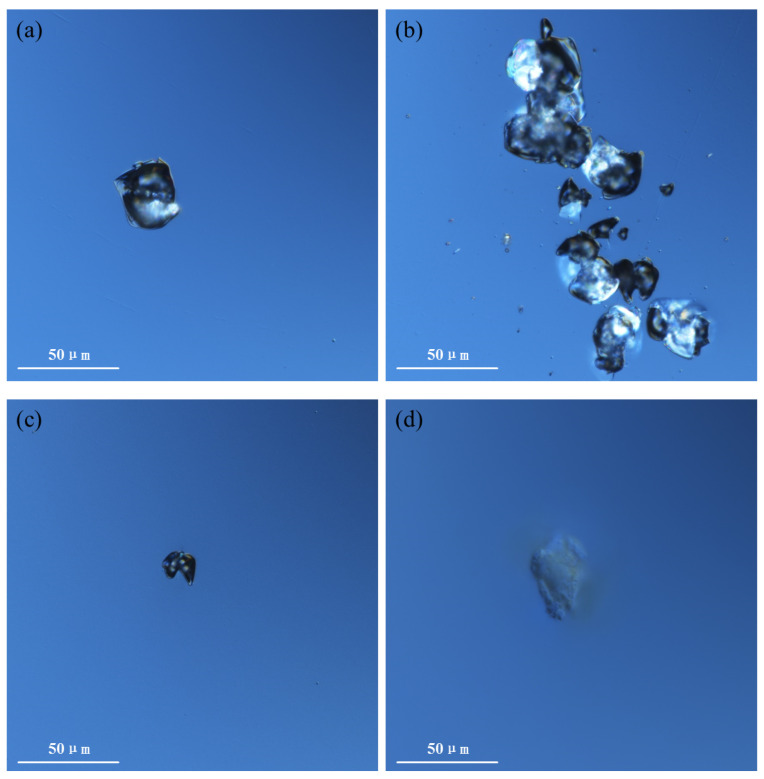
Comparison of damage pit morphology between laser-polished samples and conventional polished and etched samples. (**a**,**b**) Conventional polished and etched sample; (**c**,**d**) Laser-polished sample.

Fused silica samples processed using the optimized laser polishing scheme were compared with conventional polished and etched samples in terms of damage performance. The results are shown in Figure 12, Figure 13 and Figure 14.

Figure 12 shows the comparative test results of the LIDT between laser-polished samples and conventional polished and etched samples. From the test results, it can be seen that the LIDT of the laser-polished samples is 35.75 J/cm^2^, while that of the conventional polished and etched samples is 29.79 J/cm^2^. The LIDT of the laser-polished samples is approximately 1.2 times that of the conventional polished and etched samples.

Figure 13 shows the comparative test results of damage density between laser-polished samples and conventional polished and etched samples. From the figure, it can be seen that compared to conventional polished and etched samples, the laser-polished samples exhibit a 76.4%~90.8% reduction in damage density. Under high fluences (>25 J/cm^2^), the damage density of laser-polished samples is an order of magnitude lower than that of conventional polished and etched samples. At comparable energy densities, when the damage density of laser-polished samples is 20.83 cm^−2^, the damage density of conventional polished and etched samples is 226.49 cm^−2^.

Figure 14 shows the comparative results of damage pit morphology between laser-polished samples and conventional polished and etched samples. The damage pit morphology of conventional polished and etched samples primarily consists of single-point damage pits (Figure 14a), accompanied by a small number of concentrated linearly distributed damage pits (Figure 14b), which are speculated to be related to residual subsurface cracks in the components. The damage pit morphology of laser-polished samples, however, primarily consists of single-point damage pits (Figure 14c) and single-point superficial damage (Figure 14d). Compared to conventional polished and etched samples, the damage pit morphology of laser-polished samples changes significantly, and there are basically no concentrated linearly distributed damage pits caused by residual cracks, indicating that laser polishing can more effectively remove subsurface cracks in fused silica components compared to conventional polishing and etching.

Combining Figure 12, Figure 13 and Figure 14, it is evident that the damage performance of laser-polished samples is significantly superior to that of conventional polished and etched samples. Compared to conventional polishing and etching, the optimized laser polishing process can more effectively achieve the removal of subsurface cracks through laser fusion, thereby enhancing the damage performance of fused silica samples.

## 4. Conclusions

This study investigates the damage performance of CO_2_ laser-polished fused silica by analyzing the effects of various factors and comparing it with that of conventional polished and etched samples. The main conclusions are as follows:

(1) In terms of damage performance optimization, the analysis results of the influence of pre-treatment processes and laser processing parameters during CO_2_ laser polishing on the damage performance of fused silica samples indicate that the improvement in the damage performance of laser-polished samples is directly related to the removal of surface contaminants and the fusion of subsurface cracks. The pre-treatment process primarily involves cleaning and etching to eliminate surface contaminants and expose subsurface cracks, while the laser processing technique employs laser fusion to achieve molten healing of these subsurface cracks. Therefore, to obtain laser-polished fused silica samples with optimized damage performance, the pre-treatment stage must maintain standard cleaning procedures, appropriate etching, and a clean water environment. Concurrently, the laser processing stage requires establishment of an appropriate thermal melting temperature to maximize subsurface crack fusion without inducing ablation. For instance, in the laser polishing of a φ50 mm × 5 mm component, standard ultrasonic cleaning and HF etching are first performed in a clean water environment. Subsequently, based on temperature control requirements, the laser power is adjusted to 16~24 W, thereby obtaining a laser-polished sample with optimized damage performance.
(2) Regarding the validation of damage performance, the comparative results of damage performance between optimized laser-polished samples and samples treated with conventional polishing and etching demonstrate that the damage performance of laser-polished samples is significantly superior to that of conventional polished and etched samples. The LIDT of the laser-polished samples can reach 1.2 times that of the conventional polished and etched samples, and the damage density under high fluences (>25 J/cm^2^) can be reduced by more than an order of magnitude compared with the conventional polished and etched samples.

By analyzing the influence of various factors on the damage performance of laser-polished samples, this research has established an optimized laser polishing technical scheme. It confirms the definitive role of CO_2_ laser polishing in enhancing the laser damage resistance of fused silica samples, thereby providing technical support for the development of next-generation optical manufacturing technologies.

## Figures and Tables

**Table 1:** Water sample quality test results.

Water Sample	Conductivity (25 °C)	ICP-OES Elemental Analysis (Compared with Ultra-Pure Water)	ICP-MS Elemental Analysis (Compared with Ultra-Pure Water)
ultra-pure water	0.0055 mS/m	—	—
water sample1	0.064 mS/m	higher Si content	no significant difference
water sample2	0.008 mS/m	higher Ca content	higher Ti, Mn, Sr, Sn, Ba content

## Data Availability

The data presented in this study are available on request from the corresponding author. The data are not publicly available due to the data also forming part of an ongoing study.

## Abbreviations

The following abbreviations are used in this manuscript:

LIDR	Laser-Induced Damage Resistance
SEM	Scanning Electron Microscope
AFM	Atomic Force Microscope
LIDT	Laser-Induced Damage Threshold

## References

[1] Jiang L.W., Chi H.L., Xu Y.Y., Tao H.M. (2020). Current status and development prospects of the fused quartz industry. Chin. Mark..

[2] Li Y.G., Yuan Z.G., Wang J., Xu Q. (2017). Laser-induced damage characteristics in fused silica surface due to mechanical and chemical defects during manufacturing process. Opt. Laser Technol..

[3] Miller P.E., Bude J.D., Suratwala T.I., Shen N., Laurence T.A. (2010). Fracture-induced subbandgap absorption as a precursor to optical damage on fused silica surfaces. Opt. Lett..

[4] Suratwala T.I., Miller P.E., Bude J.D., Steele W.A., Wong L.L. (2011). HF-based etching processes for improving laser damae resistance of fused silica optical surfaces. J. Am. Ceram. Soc..

[5] Manes K.R., Spaeth M.L., Adams J.J., Bowers M.W., Bude J.D., Carr C.W., Yang S.T. (2016). Damage mechanisms avoided or managed for NIF large optics. Fusion Sci. Technol..

[6] Neauport J., Lamaignere L., Bercegol H., Pilon F., Birolleau J.C. (2005). Polishing-induced contamination of fused silica optics and laser induced damage density at 351nm. Opt. Express.

[7] Campbell J.H., Suratwala T.I. (2000). Nd-doped phosphate glasses for high-energy/high-peak-power lasers. J. Non-Cryst. Solids.

[8] Zhao L.J. (2022). Study on CO_2_ Laser Polishing Mechanism and Removal Process of Fused Silica Material. Ph.D. Thesis.

[9] Wang D., Fan F., Liu M., Tan T., Li Y. (2020). Top-hat and Gaussian laser beam smoothing of ground fused silica surface. Opt. Laser Technol..

[10] Temple P.A., Lowdermilk W.H., Milam D. (1982). Carbon dioxide laser polishing of fused silica surfaces for increased laser-damage resistance at 1064 nm. Appl. Opt..

[11] Hildebrand J., Hecht K., Bliedtner J., Muller H. (2011). Laser beam polishing of quartz glass surfaces. Phys. Procedia.

[12] Hecht K., Bliedtner J., Rost M., Muller H., Schmidt T. (2015). Carbon dioxide laser beam polishing of fused silica surfaces-process development and optimization. Adv. Eng. Mater..

[13] Weingarten C., Schmickler A., Willenborg E., Wissenbach K., Poprawe R. (2017). Laser polishing and laser shape correction of optical glass. J. Laser Appl..

[14] Cormont P., Bourgeade A., Carvaro S., Donval T., Doualle T., Gaborit G., Gallais L., Rullier J.L. (2015). Relevance of carbon dioxide laser to remove scratches on large fused silica polished optics. Adv. Eng. Mater..

[15] Luo X.Y., Yang W., Li Y.G. (2024). The influence of laser beam shaping on surface roughness, surface figure and mid-spatial frequency of fused silica glass in CO_2_ laser smoothing. Opt. Laser Technol..

[16] Xiang X., Zheng W.G. (2011). Irradiation effects of CO_2_ laser parameters on surface morphology of fused silica. Chin. Phys. B.

[17] Zhao L.J., Cheng J., Chen M.J., Yuan X., Liao W., Liu Q., Yang H., Wang H. (2019). Formation mechanism of a smooth, defect-free surface of fused silica optics using rapid CO_2_ laser polishing. Int. J. Extrem. Manuf..

[18] He T., Wei C.Y., Jiang Z., Zhao Y., Shao J. (2018). Super-smooth surface demonstration and the physical mechanism of CO_2_ laser polishing of fused silica. Opt. Lett..

[19] Nowak K.M., Baker H.J., Hall D.R. (2015). Analytical model for CO_2_ laser ablation of fused quartz. Appl. Opt..

[20] Doualle T., Hebert D., Combis P., Hecquet C., Gallais L., Rullier J. (2016). Comparison between fused silica of type II and III after surface heating with a CO_2_ laser. Appl. Phys. A Mater. Sci. Process..

[21] He T., Wei Z.Y., Jiang Z.G., Yu Z., Chao Z., Shao J.D. (2018). Numerical model and experimental demonstration of high precision ablation of pulse CO_2_ laser. Chin. Opt. Lett..

[22] Jiang Y. (2012). Theoretical and Experimental Study on Surface Damage Repair of Fused Silica Optical Components. Ph.D. Thesis.

[23] Tan C., Zhao L.J., Chen M.J., Cheng J., Zhang Y., Zhang J., Yang H., Yin Z.Y. (2022). Repaired morphology of CO_2_ laser rapid ablation mitigation of fused silica and its influence on downstream light modulation. Sci. China (Technol. Sci.).

[24] Jiang Y., Xiang X., Liu C.M., Luo C.S., Wang H.J., Yuan X.D., He S.B., Ren W., Zheng W.G. (2012). Two localized CO_2_ laser treatment methods for mitigation of UV damage grouth in fused silica. Chin. Phys. B.

[25] Shen X., Song C., Shi F., Tian Y., Tie G., Qiao S., Peng X., Zhang W., Hou Z. (2023). Research on Laser-Induced Damage Post-Restoration Morphology of Fused Silica and Optimization of Patterned CO_2_ Laser Repair Strategy. Micromachines.

[26] Tan C., Zhao L.J., Chen M.J., Cheng J., Yang H., Liu Q., Yin Z.Y., Ding W.Y. (2022). Morphology evolution mechanisms and localized structural modification of repaired sites on fused silica optics processed by CO_2_ laser rapid ablation mitigation. Opt. Laser Technol..

[27] Luo X.Y. (2022). Study on the Evolution of Full-Bandwidth Surface Errors in Fused Silica Components Processed by CO_2_ Laser Polishing. Master’s Thesis.

[28] Lu G., Li X., Yan D., Wang D., Peng Y., Wang K. (2021). Influence of parameters on the surface roughness of the CO_2_ laser polished fused silica glass. J. Light Electronoptic.

[29] Adamčík L., Igaz R., Štefančin L., Kubovský I., Kminiak R. (2023). Evaluation of the Surface Irregularities of the Cross-Section of the Wood after CO_2_ Laser Cutting. Materials.

[30] Wang H.J., Li X.B., Lv H.B., Yuan X.D., Zheng W.G. (2010). Effect of CO_2_ Laser Pretreatment Parameters on the Surface Roughness of Quartz Substrates. High Power Laser Part. Beams.

